# Accounting for Emotional Value: A Review in Disability Organizations

**DOI:** 10.3389/fpsyg.2021.741897

**Published:** 2021-09-24

**Authors:** Pilar Tirado-Valencia, Silvia Ayuso, Vicente Fernández-Rodríguez

**Affiliations:** ^1^Department of Finance and Accounting, Universidad Loyola Andalucía, Córdoba, Spain; ^2^Mango Chair in Corporate Social Responsibility, ESCI-UPF, Pompeu Fabra University, Barcelona, Spain; ^3^Department of Management, Universidad Loyola Andalucía, Córdoba, Spain

**Keywords:** emotional value, social accounting, disability, emotional well-being, social impact, quality of life

## Abstract

The aim of this paper is to examine how disability organizations account for the emotional value they create for their stakeholders. Based on a review of the literature on emotional value measurement in third sector organizations working in the disability sector, we investigate to what extent emotional value is considered in their social accounting process and what type of value variables, indicators and proxies are used. The results reveal that the analysis of some quality of life domains provides appropriate evidence to represent the emotional value generated by these organizations but that there is a great dispersion in applied instruments and methodologies. The study improves the knowledge and understanding of existing approaches to capture the emotional component of social value creation and contributes to its standardization. Our analysis has implications for the management of disability organizations that can use social accounting to evaluate their performance and improve their effectiveness and efficiency, showing a more complete picture of the social value generated. Likewise, it can be an instrument to make the contribution and social benefits of these organizations visible in all their breadth, improving transparency and legitimacy.

## Introduction

Social value measurement contributes to improve the management of non-profit organizations and social enterprises and has attracted the attention of researchers and social sector leaders, concerned with demonstrating the role of third sector entities in solving complex social problems, such as inequality, exclusion or poverty (Ebrahim and Rangan, 2010, 2014; Cordery and Sinclair, 2013; Luke et al., 2013; Millar and Hall, 2013; Arvidson and Lyon, 2014; Grieco et al., 2015; Corvo et al., 2021).

Compared to the use of other traditional performance measurement methodologies based on cost-effectiveness and cost-benefit analysis, the study of social value creation allows evaluating the transformative capacity of social organizations in all its breadth (Matthews, 2015). This value transcends the economic-financial market criteria of traditional financial statements (Mook et al., 2003; Richmond et al., 2003; Retolaza and San-Jose, 2021) and is essential to demonstrate the social contribution and progress toward the fulfillment of the mission of third sector entities (Sawhill and Williamson, 2001; Ebrahim and Rangan, 2014). Furthermore, in an environment of crisis and austerity, social value measurement can be a valid instrument of institutional legitimation for entities that are dependent on financial aid (Bagnoli and Megali, 2011; Luke et al., 2013; Mäkelä, 2021), justify the efficient use of the funds received (Millar and Hall, 2013; Arvidson and Lyon, 2014; Ebrahim and Rangan, 2014; Mook et al., 2015; Lazkano and Beraza, 2019; Ruiz-Lozano et al., 2020) and help to improve the performance of social organizations (Ebrahim and Rangan, 2010, 2014; Gibbon and Dey, 2011; Cordery and Sinclair, 2013; Grieco et al., 2015; Mook et al., 2015). Particularly, in the field of organizations that work with people with disabilities, an important part of the social value is generated by its impact on improving the quality of life (QOL) and the emotional well-being of both service users and other stakeholders such as families, caregivers and staff (Owen et al., 2015).

However, although quality of life indicators are quite frequent in health economics studies (Edwards et al., 2013), and more specifically in areas such as disability (Jones et al., 2016; Moral Torres et al., 2020), so far there is no accepted model of emotional value accounting for disability organizations. This entails the risk of relegating the organizations' contribution to improving the emotional well-being of its stakeholders to the background or even render it “invisible” (Fuertes-Fuertes et al., 2020; Retolaza and San-Jose, 2021).

The problem is that the transfer of emotional value is difficult to quantify and monetize and thus limits the possibility of offering a complete vision of the benefits created by disability organizations (Grieco et al., 2015; Retolaza and San-Jose, 2021). To face this difficulty, studies are needed that present empirical evidence on the variables of the non-financial value generated and that allow consensus on standardized metrics, facing discretion in quantifications and allowing the generalization of the results (Arvidson and Lyon, 2014; Farr and Cressey, 2019; Jones et al., 2020; Retolaza and San-Jose, 2021).

The aim of this paper is to examine how disability organizations account for the emotional value they create for their stakeholders. Based on a review of the literature on emotional value measurement in third sector organizations working in the disability sector, we investigate to what extent emotional value is considered in their social accounting process and how it is addressed, i.e., which stakeholders are considered and what type of value variables, indicators and proxies are used. Thus, the study improves the knowledge and understanding of existing approaches to capture the emotional component of social value creation and contributes to its standardization.

The paper is structured as follows: after this introduction, the second section analyzes the contribution of social accounting to the value analysis and performance assessment of third sector entities in general. Section three highlights the importance of measuring emotional value for disability organizations and analyzes some contributions to the assessment of quality of life. Section four describes the methodology used and the fifth section describes the results of our study. Finally, section six discusses the results obtained and presents the main conclusions.

## Social Accounting in Social Organizations

Cost-effectiveness and cost-benefit analyzes have been frequently used by social entities to evaluate their interventions (Krlev et al., 2013). However, these methodologies have been widely criticized for not being able to reflect the multitude of intangible benefits derived from the strong mission of social entities (Ebrahim and Rangan, 2010, 2014; Bagnoli and Megali, 2011; Gibbon and Dey, 2011; Arvidson et al., 2013; Grieco et al., 2015; Mäkelä, 2021). In addition, these methodologies do not have a holistic vision of that allows visualizing all the changes generated by the interventions, nor do they take into account the long-term effects (Edwards et al., 2013; Banke-Thomas et al., 2015). In short, cost-effectiveness and cost-benefit analyzes do not offer a complete vision of the value created by social organizations that helps them to understand what changes for stakeholders and how they can be able to improve their performance (Sawhill and Williamson, 2001; Ebrahim and Rangan, 2010, 2014; Cordery and Sinclair, 2013; Grieco et al., 2015; Mook et al., 2015).

Third sector organizations not only need to incorporate social benefits in their measurement systems, but also to evaluate compliance with the expectations of their stakeholders (Gibbon and Dey, 2011; Luke et al., 2013; Costa et al., 2014; Grieco et al., 2015; Mäkelä, 2021). This requires to analyze the social value creation in a context that differs from that of commercial companies operating in the market, and at different levels including individuals (beneficiaries, family members, employees and volunteers), organizations, community and wider society (Becker, 2001; Mook et al., 2003; Luke et al., 2013).

To analyze and evaluate the social value generated for stakeholders, organizations design social accounting systems (Richmond et al., 2003; Ayuso et al., 2020; Corvo et al., 2021; Retolaza and San-Jose, 2021). Richmond et al. (2003) define social accounting as “a systematic analysis of the effects of an organization on its communities of interest or stakeholders.” This social accounting includes value transfers to stakeholders that do not originate in a market transaction, some of which have an emotional nature (Retolaza et al., 2016).

There are different proposals for the systematization of social accounting: social balance (Vaccari, 1997), common good balance (Felber, 2008), blended value accounting (Emerson, 2003), expanded value-added statement (Meek and Gray, 1988), integrated social value (Retolaza et al., 2016) or social return on investment (SROI) (Nicholls et al., 2009). However, despite the diversity of proposals, several authors have highlighted three essential aspects shared by social accounting systems: engagement and prioritization of stakeholders (Hall et al., 2015; Costa and Pesci, 2016), the search for evidence on the social value generated (Arvidson et al., 2013; Millar and Hall, 2013) and the quantification and monetization of social value (Mook et al., 2003; Richmond et al., 2003; Gibbon and Dey, 2011; Banke-Thomas et al., 2015).

In third sector organizations, social accounting must fulfill two major functions. On the one hand, it is a complex, dynamic, multidimensional and multi-stakeholder accounting system (Costa et al., 2014; Mäkelä, 2021) that holds a humanistic vision of the value created by the entity, in order to improve the well-being of people and face social challenges (Aguado et al., 2015). On the other hand, it must be an instrument that favors improvements in internal management (Ebrahim and Rangan, 2010, 2014; Gibbon and Dey, 2011; Ormiston, 2019; Mäkelä, 2021) and organizational legitimation (Gibbon and Dey, 2011; Luke et al., 2013; Lazkano and Beraza, 2019; Retolaza and San-Jose, 2021). In addition, Mäkelä (2021) argues that the social accounting of these entities should be an instrument for socialization, and not a purely organizational phenomenon that responds to pressures from funders.

These objectives can be addressed in different ways when approaching the social accounting process (Brown and Dillard, 2015; Mäkelä, 2021), generating different theoretical paradigms regarding the evaluation of interventions and the measurement of social value (Edwards et al., 2013). On the one hand, it is necessary to develop an opening-up process that emphasizes stakeholder participation and engagement, since their testimony confers credibility and rigor to the measurements. Stakeholders acquire a leading role in social accounting, which becomes a dialogical and narrative instrument. On the other hand, a more technical-rational approach maintains a closing-down approach where performance measurement, accountability, transparency and control become important objectives of the accounting system (Ormiston, 2019). Both paradigms are complementary and necessary for social accounting to meet its objectives.

Another issue related to the social accounting of non-profits and social sector entities is its relevance from a public value perspective, in a context of public policy reforms (Millar and Hall, 2013). Measuring the social value of non-profit entities makes it possible to legitimize the allocation of public resources and the grants-in-aid (Matthews, 2015), and is useful when analyzing the impacts of public policies in areas such as disability (Farr and Cressey, 2019). Social value measurement reveals which organizations are the ones that contribute more effectively and efficiently to create public value (Fuertes-Fuertes et al., 2020).

Ormiston (2019) concludes that there are multiple rationalities in the social value literature. The multifaceted nature of social value and its study from different approaches has turned it in an interdisciplinary issue whose benefits can be highlighted from different organizational functions. Furthermore, the existence of an emotional component as part of the social value generated by organizations requires its study in transdisciplinary terms. However, despite the weight of emotional factors in creating social value, there are still few studies that have analyzed the role of emotional value in improving the management of organizations, or that link the social accounting of third sector entities with other disciplines such as psychology or mental health.

The lack of consensus on how to measure the social value generated by third sector organizations (Arvidson and Lyon, 2014), together with the absence of studies that allow systematically to assess emotional value, has led us to consider the need to investigate the mechanisms for the recognition of this value. Specifically, we will focus on the sector of disability organizations, as this area has attracted a good deal of research on social impacts (Krlev et al., 2013). In addition, it is a sector that works to improve the quality of life of people, so the emotional component is part of its social mission and has an important weight in its interventions.

## Social Accounting and Emotional Well-Being in Disability Organizations

The concept of emotional value differs depending on the type of organization. In commercial firms, the dimensions of emotional value could be related to the consumers perspective in their market transactions, and could be quantified applying methodologies from the field of service quality, or to the fulfillment of workers' labor expectations and their commitment to the company (Ruiz-Roqueñi, 2020). However, for non-profit organizations, especially those that work with groups of disadvantaged people such as the disabled, emotional value is associated with improvements in the quality of life and the well-being, as well as with the consolidation of public collective values such as equality, inclusion or dignity (Aguado et al., 2015; Farr and Cressey, 2019). Since these issues are related to the fulfillment of the mission of nonprofit organizations, it is essential that they are reflected in its social accounting model (Akingbola et al., 2015). However, it is not easy to account for these values (Edwards et al., 2013; Millar and Hall, 2013; Banke-Thomas et al., 2015; Willis et al., 2018; Ashton et al., 2020).

In organizations in the disability sector, emotional value is created in multiple ways: taking care of the physical and mental health of its beneficiaries, improving their social relationships, guiding families and freeing up time for leisure and rest, or creating employment opportunities for users and their families (Owen et al., 2015; Jones et al., 2020; Barba-Sánchez et al., 2021). For the people who work in these organizations there is also an emotional plus due to the satisfaction of being able to help people in need. At the community level, emotional value is related to awareness and sensitization of the problems associated with disability, and also with the change in attitude of society toward disabled people (Owen et al., 2015; Farr and Cressey, 2019).

From a social accounting perspective, this value could be used for multiple purposes in the management of organizations in the disability sector, including setting strategies (Schalock et al., 2018), improving effectiveness and efficiency (Edwards et al., 2013; Golics et al., 2013; Owen et al., 2015; Onyx et al., 2018; Jones et al., 2020), continuous improvement of intervention programs (Van Loon et al., 2013; Willis et al., 2018), project evaluation and the choice of alternatives (Onyx et al., 2018; Willis et al., 2018; Hutchinson et al., 2020), internal and external communication (Shaw, 2018), or justification of the funds received (Akingbola et al., 2015; Owen et al., 2015; Farr and Cressey, 2019; Jones et al., 2020; Ruiz-Lozano et al., 2020).

The construction of a social accounting model that integrates emotional value begins with the identification of the value variables for each stakeholder (Retolaza et al., 2015). The main stakeholders of a disability organization are its users who participate and benefit from the interventions and programs and experience improvements in their quality of life. Among the most relevant scientific works in the conceptualization and measurement of quality of life we find those of Schalock and Verdugo (2002) and Schalock et al. (2008). They propose a multidimensional model of quality of life that could serve as a starting point in the delimitation of the corresponding value variables. Their model is structured around eight dimensions, grouped into three factors: (a) independence: personal development, self-determination, (b) social participation: interpersonal relations, social inclusion, rights, and (c) well-being: emotional well-being, physical well-being, material well-being.

The emotional well-being is associated with the psychological aspects of the person and is related to positive feelings such as self-acceptance, empathy, autonomy, purpose in life or personal growth (Ryff and Keyes, 1995). Emotional well-being is thus differentiated from well-being arising from the individual's state of health and from well-being providing the necessary means to lead a dignified life, although there are obvious links between all these well-being dimensions. Accordingly, for disability organizations to improve people's emotional well-being, it is not enough with a welfare model that prolongs people's life expectancy (Jones et al., 2020), or that is focused only in improving their physical and mental state and the coverage of basic needs (Ashton et al., 2020). Overcoming this neoclassical approach, it is necessary for these organizations to act in other quality of life domains, such as those related to security, trust and the affective environment. The development of individual and collective capacities constitutes an effective strategy for this (Edwards et al., 2015). Regarding individual capacities, it will be necessary to promote social skills in order to improve interpersonal relationships, in addition to reinforcing the skills needed to be able to lead an autonomous life. These improvements are achieved through programs that promote employability (e.g., Owen et al., 2015; Jones et al., 2020), entrepreneurial competences (e.g., Barba-Sánchez et al., 2019, 2021; Ortiz García and Olaz Capitán, 2021), learning (e.g., Shaw, 2018), or participation in artistic, cultural or recreational activities (e.g., Onyx et al., 2018; Bosco et al., 2019). Regarding collective capacities, it will be necessary to consider issues related to equity (Ashton et al., 2020) and dignity (Aguado et al., 2015), and to sensitize society about social problems (Edwards et al., 2015).

**Figure 1:**
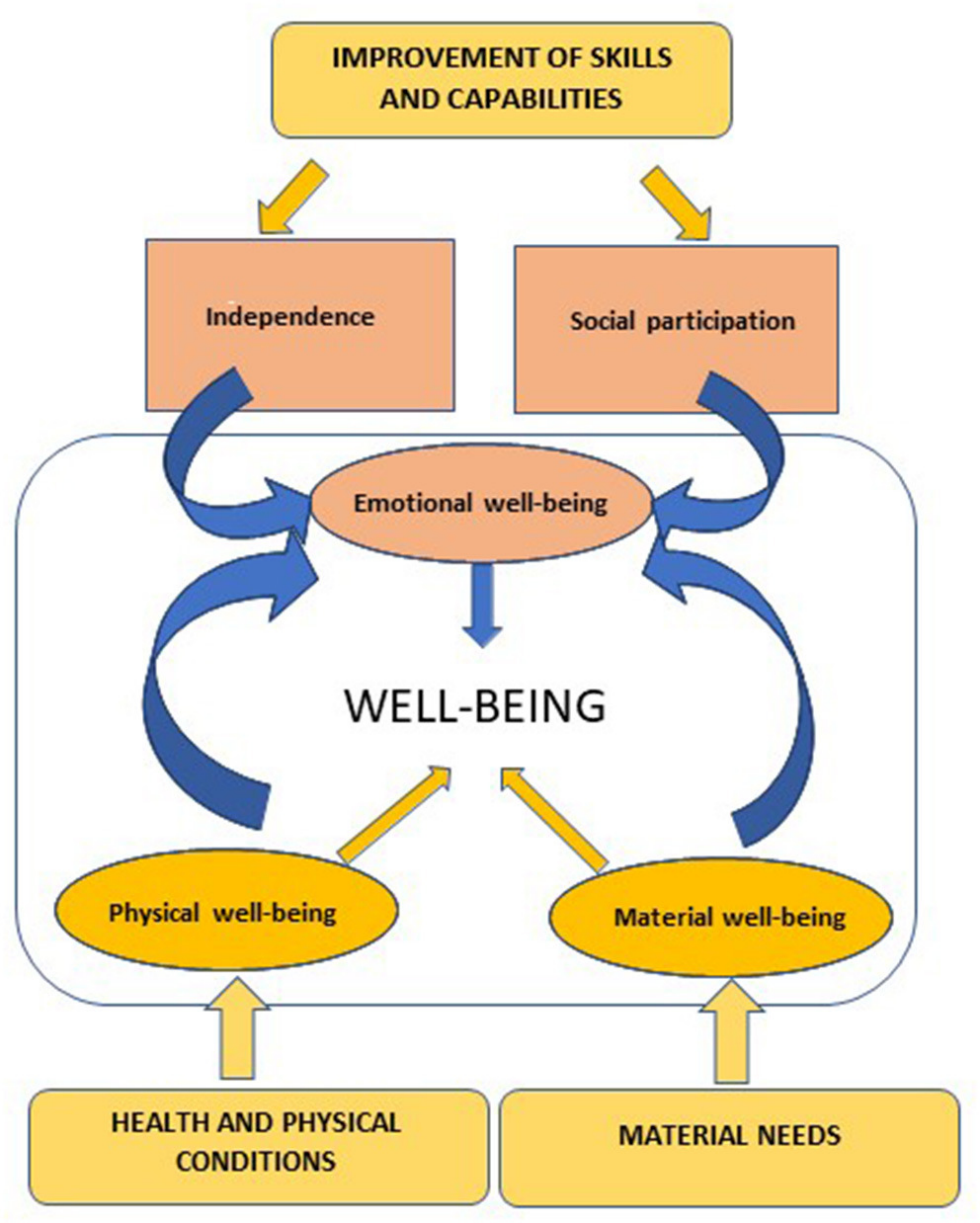
Emotional value and quality of life.

In conclusion, emotional value variables would be linked to psychological aspects of the person's well-being (independence, confidence, self-esteem, etc.) conditioned, on the one hand, by their health situation and the satisfaction of their basic needs, and on the other hand, by the state of their social and family relationships. In addition, emotional value would be influenced by the improvement of skills and capabilities that allow people with disabilities to achieve full integration. Figure 1 illustrates this relationship between the quality of life dimensions proposed by Schalock and Verdugo (2002) and how they are influenced by the diverse support services offered by disability organizations.

However, disabled users or participants or organizational interventions and programs are not the only stakeholders experiencing effects on their emotional well-being. Since social accounting is a multi-stakeholder accounting system, it should consider the changes experienced by an array of groups that interact with users/participants, starting with their family members and caregivers, and continuing with the workers of the different organizations involved in the interventions and programs, or even citizens with whom they may share different activities. In this sense, the identification of the value variables of emotional well-being from the point of view of the beneficiary would be complemented with an organizational and community perspective (Schalock et al., 2011).

One of the main problems of measuring social value that also applies to disability organizations is the search for evidence for its recognition and quantification (Arvidson et al., 2013; Millar and Hall, 2013). Due to its intangible nature, the measurement of well-being requires indicators based on a subjective assessment by the individual (Ruiz-Roqueñi, 2020). However, in the field of disability, there is a great diversity of questionnaires and standardized quality of life scales that help to measure emotional value variables (Townsend-White et al., 2012). These scales have traditionally been used to design individualized intervention plans for social entities that work in the field of disability (Schalock et al., 2008), and thus enjoy considerable consensus in the field of psychology. One of the most widely used questionnaires is the Quality of Life Questionnaire (QOL-Q), which has been validated in numerous studies (e.g., Schalock et al., 2005).

This measurement approach seems potentially interesting for capturing the emotional value in disability organizations. Indicator items associated with each quality of life domain are used to assess either the person's perceived well-being on the item (“self-report”) or an objective indication of the person's life experiences and circumstances (“direct observation”) (Schalock et al., 2008; Van Loon et al., 2013). These indicators not only allow to make a clinical diagnosis that determines the most appropriate medical treatment for each individual, but also establish his or her subjective well-being (Diener et al., 1999), when evaluating other aspects such as life satisfaction, positive effects (e.g., happiness) and the absence of negative effects (e.g., sadness/worry).

Finally, a common challenge in social accounting is how to monetize the identified and quantified value in the absence of market references. Traditionally, social value monetization techniques have been based on the preference based valuation approaches that use market prices proxies (revealed preference methods), or surveys to ask individuals their willingness to pay (stated preference methods). However, these preferences are not always consistent and reliable in behavioral and emotional economics studies (Fujiwara, 2014). Instead, Fujiwara (2014) proposes the use of methodologies to measure welfare, life satisfaction and happiness based on well-being valuation. This well-being valuation approach estimates the impact of the good or service and income on people's subjective well-being and uses these estimates to calculate the exact amount of money that would produce the equivalent impact on well-being. Some other useful measurement tools from the point of view of public health economics as the quality adjusted life years (QALYs), the disability adjusted life years (DALYs), the healthy life-years gained, the caregivers CAREQOL or the EuroQOL can also be relevant in the field of disability (Edwards et al., 2013).

## Methods

To identify the most relevant studies that analyze the emotional value generated by third sector organizations working in the disability sector, we conducted a systematic review of the academic literature in the field. Our literature review followed a PRISMA approach (Moher et al., 2015) to identify the relevant items to carry out the review and define the eligibility criteria for selecting the studies. Since previous literature reviews of social value studies have identified a relatively small number of studies from academic journals (e.g., Banke-Thomas et al., 2015; Hutchinson et al., 2018), we have extended our review to gray literature to search for available reports from disability organizations that have applied social accounting processes.

### Search Strategy

The search was comprised of two main steps: first we identified academic peer-reviewed articles, and second, gray literature reports. For peer-reviewed articles, we searched Web of Science, Scopus, PubMed and MedLine databases in February 2021. Terms used were “disabilit^*^ AND social accounting,” “disabilit^*^ AND social value,” “disabilit^*^ AND social impact,” “disabilit^*^ AND social return on investment,” and “disabilit^*^ AND SROI.” These search terms were applied to the title, abstract and keywords fields. All searches were repeated replacing the term “disabilit^*^” with “mental^*^.” We searched for articles published in English from 2010 onward, which is when studies on social value and impact measurement started to gain importance (Krlev et al., 2013). Once the repetitions were eliminated, we obtained a total of 185 academic papers were obtained.

For gray literature, we searched on the website of Social Value UK (https://socialvalueuk.org) whose database has been considered one of the best repositories of reports on social value analysis in third sector organizations (Hutchinson et al., 2018). In April 2021 we found 45 reports classified within the topic “disability” in the search engine of its website.

Two researchers independently conducted the search and reviewed all retrieved records. Studies identified through the searches were screened by reading the abstracts or executive summaries. Agreement was reached regarding the final eligibility based on the set inclusion and exclusion criteria.

### Inclusion and Exclusion Criteria

We included peer-reviewed and gray literature studies that dealt with emotional value measurement in disability organizations. We included only studies dealing explicitly with disability. Thus, studies related to the loneliness of the elderly or the treatment of addictions were discarded, although these problems could be the cause of some disabilities.

Regarding the type of activity carried out by the organizations, we incorporated both entities directly dedicated to improving the health of disabled people, as well as those that perform activities that indirectly affect the welfare of the disabled. For example, we included studies that analyze the impacts generated by social entities dedicated to improving employment opportunities or artistic activities.

Nevertheless, we excluded all those studies that were not related to the management of organizations in the disability sector, such as studies that analyzed clinical aspects, treatment of diseases associated with disability, validation of measurement scales of quality of life and public health. Likewise, we discarded all those papers and reports that focused on the study of health economics, health costs or economic impacts of public and private health interventions. Finally, we excluded all articles that were merely theoretical or conceptual.

In the case of the disability-related reports from the Social Value UK database, we included only those reports that described in detail the steps undertaken to measure emotional components of social value and excluded all publications that were only summaries of more extensive reports, and all documents that were not available in English.

After this process, we finally identified a total of 29 studies related to the aim of our study: 10 academic papers and 19 reports from the Social Value UK database. The studies are listed in the Appendix with consecutive numbers from 1 to 29.

### Data Extraction and Synthesis

We extracted information from each study and classified data in two pre-developed summary tables. The first one captured the basic study characteristics such as type of organizational activity, type of disability addressed by the organization, reference year(s) for conducted social value measurement, method used for social value measurement, stakeholders included in study, purpose of the study from the organizational perspective, stated organizational learnings and reported limitations. The second summary table was aimed at recording the information about the prominent elements in the social value measurement process and included type of emotional outcomes (value variables) identified, type of indicators used, type of financial proxies used, type of adjustment factors used (deadweight, displacement, attribution and drop-off) and performance of a sensitivity analysis.

Afterwards, in order to classify the emotional value variables, indicators and proxies found in the different studies, we built categories in an iterative process. The findings are summarized in Tables 1, 2 in the next section.

**Table 1:** Disability organizations' stakeholders that receive social value.

**Stakeholder**	**Description**
Users/participants	People with disabilities who are targeted by the organizational interventions and programs and who are their direct beneficiaries
Families/carers	Family members and professional carers who provide care to users/participants and whose relationship will change due to the activities undertaken by the organization
Staff	Employees of organizations that implement interventions and programs for people with disabilities or provide support, both in capabilities development and employability
Volunteers	Persons who support and enhance the service delivered by the organization on a voluntary basis
Community/society	Citizens with whom the rest of the stakeholders interact or who sporadically participate in the organization's activities
Institutions/companies/ organizations	Public and private entities that implement interventions and programs, provide support, provide funds or are clients

**Table 2:** Elements of emotional value measurement.

**Stakeholder**	**Valued outcome**	**Reference studies**	**Type of indicators used**	**Type of financial proxies used**
Users/Participants	Feeling more independent/control over their life/self-determination	2, 3, 6, 8, 9, 11, 13, 15, 16, 17, 20, 21, 22, 24, 25, 28	Self-reported level of perceived change (13, 20, 22, 24, 25); increased participation in domestic life and traveling (2, 9, 11, 24); change in DEMQOL Q13 (16)	Cost of counseling sessions or courses to develop personal autonomy (1, 28); cost of renting a one bedroom flat (13); cost of a life insurance payout (15); cost of a special education technical assistant (25); cost of additional living expenses (9); feel in control: HACT Social Value Bank (16)
	Increase in confidence	1, 3, 8, 9, 15, 16, 17, 18, 20, 21, 22, 23	Self-reported level of perceived change (8, 17, 20, 22); change in DEMQOL Q5 (16)	Cost of confidence building course (8, 15); cost of therapy (1); HACT social value bank: high confidence (16)
	Increase in self-esteem	1, 8, 13, 15, 16, 18, 21, 22, 27	Self-reported level of perceived change (1, 8, 13, 22); change in DEMQOL Q5 (16)	Cost of counseling sessions or self-esteem building course (1, 6, 8, 27); HACT social value bank: high confidence (16)
	Self-regulation of behaviors	17, 25, 26	Self-reported level of perceived change (17, 25)	Cost of counseling sessions (17, 25)
	Improvement in family/social relations and decrease in feeling isolated	1, 2, 7, 8, 9, 11, 13, 15, 16, 17, 20, 21, 22, 23, 24, 26, 28, 29	Self-reported level of perceived change (13, 16, 24); new contacts and social network participation (2, 8, 11, 17, 21, 22); assessment using loneliness scale (11)	Cost of attending day support and recreation programs (9, 13, 24, 28); cost of depression treatment (29); HACT Social Value Bank: feel belonging to neighborhood (16)
	Improvement in purpose and life satisfaction/Feeling happy	4, 8, 9, 11	Self-reported level of perceived improvement in life satisfaction (8, 9, 11)	Health-Adjusted Life Years metrics: QALYs or Disability-Adjusted Life Years (DALY) or Value of Statistical Life Year (VOSLY) (4, 9); cost of counseling sessions (8, 9)
	Feelings of dignity, respect, involvement, inclusion	6, 7, 11, 15, 20, 28	Self-reported level of perceived change (6, 28); index of community involvement (11)	Value of a donation (6); cost of diversity and disability awareness training (15, 28)
Families/carers	Reduction in worry, stress and/or anxiety	3, 5, 8, 9, 11, 13, 14, 19, 24, 26, 27, 28, 29	Self-reported level of perceived change (3, 8, 9, 13, 14, 19, 24, 26, 27, 28, 29); data from the literature about decreased probability of suffering mental health issues (5); assessment using General Health Questionnaire (EQ-5D) (11)	Cost of physical and mental treatment services to achieve the same effect (3, 8, 9, 14, 24, 26, 27, 28); cost of medical services saved (5, 13, 29)
	Improvement in family relationships	3, 9, 10, 13, 17, 19, 21, 22, 25, 27, 28	Self-reported level of perceived change (3, 9, 10, 13, 17, 19, 21, 22, 25, 27, 28)	Cost of counseling sessions or courses to recover a family relationship (3, 9, 10, 21, 25); cost of doctor visits saved (10); partial cost of bringing up a child (19); average family spend on social activities, recreation, culture or holidays (13, 17, 27, 28)
	Improvement in social life	3, 9, 16	Self-reported level of perceived change (3, 9); use of validated scales (31-item DEMQOL-proxy, 19-item Approaches to Dementia questionnaire) (16)	Cost of home care (3); cost for a gym membership (9); HACT Social Value Bank: feel belonging to neighborhood (16)
	Change in attitude toward disability	16, 28	Self-reported level of perceived change (28); use of validated scales (31-item DEMQOL-proxy, 19-item Approaches to Dementia questionnaire) (16)	Cost of (disability awareness) training (16, 28)
Staff	Awareness of rights and potential of people with disabilities	3, 9, 8	Self-reported level of perceived change (3, 8, 9)	Cost of disability awareness training (8)
	Increase in confidence/morale	8, 9	Self-reported level of perceived change (8, 9)	Cost of confidence building course (8, 9); organizational costs (9)
	Increase in satisfaction and self-esteem	8, 9, 17, 22	Self-reported level of perceived change (22); reduction in number of sickness hours (8)	Cost of sickness absence (8); willingness to contribute to a similar charitable program (22)
Volunteers	Increase in confidence and self-esteem	2, 9, 17, 21	Self-reported level of perceived change (2, 9, 17, 21); reduction in number of doctor visits (2)	Cost of physical and mental treatment services to achieve the same effect (2, 21); cost of time and transport (2); cost to volunteer abroad (17); contingent valuation (9)
	Feeling engaged and contributing to society	6, 21, 25, 29	Self-reported level of perceived change (21); number of (additional) volunteering hours (6, 25, 29)	Value of hours of service offered (6); cost of an equivalent professional (25); cost to volunteer abroad (21); value of job satisfaction according to Global Value Exchange (29)
	More involvement in society	2, 6, 25	Self-reported level of perceived change (2, 25); return of their time input (6)	Cost of a similar activity (25); increase in spending on recreation (2); multiplier of value of time inputted (6)
	Change in attitude toward disability	25	Self-reported level of perceived change (25)	Cost of disability awareness training (25)
Community/society	More positive interaction with people with disabilities	9	Self-reported level of perceived change (9)	Cost of course or lecture (9)
Institutions/ companies/ organizations	The emotional outcomes related to institutions or organizations have been assigned to the corresponding staff.			

## Results

The reviewed studies identify a series of stakeholders that receive social value from the organization's activity. Table 1 lists the stakeholders most commonly analyzed in the studies and summarizes their relationship with the disability organization. Besides users or participants with disabilities, studies include in their assessments families and caregivers, staff from different organizations, volunteers, local community or wider society, and institutions, companies or other organizations that actively contribute to the improvement of skills and capabilities of the disabled.

In general, most of the disability organizations analyzed collect information on the created social value by addressing directly their stakeholders through interviews, focus groups or *ad hoc* questionnaires. The use of these consultation methods is due to the need to identify and quantify the social value based on the perceptions of the stakeholders involved. It also responds to the convenience of reflecting different views on the complex problems associated with disability. All the analyzed cases follow a participatory approach that favors stakeholder engagement, where the narrative about the value sources provides the necessary rigor to confer credibility to the results (Ruiz-Lozano et al., 2020). In some studies, stakeholder participation facilitates the identification of the priority issues that add the most value or are of most concern, as well as the materiality analysis of the value variables, based on their significance or occurrence probability. However, despite the richness that this participatory process provides, it involves a significant consumption of resources. As a consequence, though all the studies recognize a wide diversity of affected stakeholders, there are few cases which report interaction with all of them.

Regarding the identification of the variables of the social value generated by disability organizations, the results of the review reflect the multidimensional and holistic nature of this value, by incorporating issues related to health, psychology, ethics, education, economy or internal management of organizations. We find value variables related to all dimensions of well-being: improving health, covering basic needs, improving skills and capabilities and diverse emotional aspects. However, while some important components of the individual's well-being such as health and care, the coverage of basic material needs and the improvement of skills and capabilities, are easy to measure in an objective manner (Van Loon et al., 2013), the emotional component has a purely subjective character as it is conditioned by the perception of each individual, which undoubtedly hinders its standardization.

This fact is not an obstacle that all the studies analyzed, reflect in some way this emotional component of the change induced by the organizational intervention, separately from the other dimensions of well-being and, with a considerable weight in the final sum of social value (see Table 2). The detailed analysis of the value variables incorporated in the reviewed studies allows us to affirm that there is considerable consensus regarding the recognition of the aspects that foster emotional well-being in disabled people. These impacts can be grouped into two categories. On the one hand, the contribution of the organization to the creation of positive feelings in the individual such as self-esteem or independence. On the other, the effects on the affective environment and the improvement in family and social relationships.

While all of the studies consider the impacts on the quality of life and the needs of people with disabilities, to a lesser extent they identify an improvement of emotional well-being for family members and caregivers and volunteers, perhaps because it has a lower relative weight in the set of social value created in the final sum of social value. Few studies analyze the impacts on the staff from different types of health and care organizations that participate in the interventions and programs for the disabled. For a part of these workers, knowing that they are involved in projects that directly contribute to improving the living conditions of people with disabilities provides significant emotional value for them. At the same time, one of the main activities of disability organizations consists in labor integration, which causes important impacts on the personnel of the companies that host or employ persons with disabilities. Both the impact on the personnel of these companies and their clients is usually left out in the studies.

Less frequent in the studies is the impact on the community and its contribution to values such as inclusion or equality. In some cases, the contribution to community well-being is recognized thanks to the improvement of the understanding of the problems associated with disability and by generating a greater commitment to these people, although at this level it is still necessary to advance in the recognition of the value variables.

With regard to the indicators used to quantify emotional value, the existence of different dimensions of quality of life, coming from different fields of knowledge, would justify the use in social accounting of tools from other disciplines, such as quality of life and life satisfaction questionnaires. These instruments offer the opportunity to measure emotional value with standardized scales that have already been validated. However, the results show that the use of this type of questionnaires is not widespread in the field of social accounting and that organizations prefer the use of surveys in which the subject declares his or her self-perception about the change experienced (Table 2). Surprisingly, standardized indices and scales from the fields of psychology or health are rarely used.

The measurement of emotional value from indicators on the self-reported level of perceived change is the norm among the studies analyzed. In some instances, the measurements are made in terms of improvement in the abilities to carry out certain daily activities such as traveling or shopping. The improvement in social relationships is measured, for example, by the number of new contacts or participation in social networks or by the cost of participation in recreation programs.

Finally, the proxies used for the monetization of the emotional value reflects an enormous dispersion (Table 2). First, many studies use proxies related to the cost of providing a similar service to that offered by the disability organization. These services mainly refer to psychological support and therapy, or participation in self-help courses that would achieve an equivalent effect on emotional well-being as the organizational activity. In the case of improving social relationships, common proxies are related to the cost of recreational programs or programs that favor the socialization of individuals. Second, some organizations have taken advantage of the existence of standardized proxy data bases such as HACT Social Value Bank or Global Value Exchange. Values from the field of health economics such as the Health-Adjusted Life Years (QALYs) or Disability-Adjusted Life Years (DALY) are rarely used.

## Discussion and Conclusions

The review of studies on social accounting of disability organizations reveals that these organizations attach great importance to emotional value when designing their accounting systems. All studies have identified emotional value variables and have linked them to the self-esteem, autonomy, trust and improvement of social relationships, although sometimes these variables are difficult to delimit, and are grouped differently and under diverse names. According to Millar and Hall (2013), these “soft” outcomes characterize the value generated by health and social care organizations, which is why organizations are striving to incorporate them into their social accounting. Furthermore, as suggested by other authors such as Golics et al. (2013), Owen et al. (2015), Farr and Cressey (2019), and Jones et al. (2020), organizations extend these impacts to other stakeholders that are related to people with disabilities as well as to the community, and do not limit themselves to analyze effects on direct beneficiaries.

The results reveal that the analysis of some quality of life domains provides appropriate evidence to represent the emotional value generated by these organizations, and offers a holistic and understandable view of the individual's well-being, as Van Loon et al. (2013) already suggested. In comparison with an emotional value measuring system based on global perceptions of consumer in their market transactions, as might be expected in a commercial company (Lazkano and Beraza, 2019; Ruiz-Roqueñi, 2020), our results show that, in disability organizations emotional value is related to personal well-being and collective values, and goes beyond the mere state of the individual's health or the coverage of their basic needs (Ashton et al., 2020; Jones et al., 2020).

The analysis shows that the processes for determining the elements of the social accounting systems of these organizations have required stakeholder engagement, whose identification and participation is an essential requirement to give legitimacy to the analysis (Arena et al., 2015; Ruiz-Lozano et al., 2020). However, this dialogue-based and deductive process should be accompanied by mechanisms that allow validating whether the variables and their quantification accurately reflect the stakeholders' perceptions about the impacts they have received. This would help to partially alleviate accusations of arbitrariness in the recognition and quantification of value variables (Millar and Hall, 2013). Furthermore, Banke-Thomas et al. (2015) suggest that in order to better reflect the complexity of social changes, stakeholders' discourse could be accompanied by the theory of change that underlies the interventions, to make more visible the links between social change dimensions and the causal relationships between the value variables. In some of the reviewed studies we have found that this reflection process has been carried out and documented.

Since social accounting is a costly process (Owen et al., 2015), some authors such as Gibbon and Dey (2011), Krlev et al. (2013), and Bosco et al. (2019) refer to the need to perform a materiality analysis of the issues that are relevant for stakeholders. This allows identifying the most significant value variables, to focus efforts on them and maximize performance. Nevertheless, this type of analysis is rare among the cases analyzed since they apparently trust that the stakeholders' report accurately reflects their priorities.

Emotional value measurement has been based on qualitative methodologies that build on the perception of the changes generated and on the fair value that stakeholders attribute to them. However, the lack of homogeneity in the measurements shows that we are still very far from the necessary standardization to guarantee the generalization and comparison of the results (Shaw, 2018; Jones et al., 2020; Ruiz-Lozano et al., 2020). This limitation is due to subjectivity in perceptions and the use of self-reported attributions to measure emotional value, conditioned by the environment and the moment in which the measurement is conducted (Farr and Cressey, 2019). The use of these measurements makes it possible to make a diagnosis of the conditions in which the subject finds himself or herself based on his or her own self-perception. However, they do not necessarily reflect the emotional value generated by the organization (Fuertes-Fuertes et al., 2020; Jones et al., 2020). For this reason, in most of the cases analyzed, the value is measured in terms of improvements or advances in people's well-being, through the use of indicators such as the self-perceived level of change.

Van Loon et al. (2013) state that another important limitation in the use of self-perception indicators is that the perception of the subjects is conditioned by personal, cultural and environmental factors. To face this limitation, many of the reviewed studies have incorporated in their social accounting counterfactual analyzes and adjustment factors that try to delimit what part of the change is attributable to the intervention of the organization and is not due to other factors unrelated to its activity.

Although some authors have suggested the use of indicators that are specific from the field of health economics to measure social value objectively, such as disability adjusted life years (DALYs) (Edwards et al., 2013), we have found few studies that use this type of indicators. Neither have we observed many studies that use standardized scales of quality of life measurement and emotional well-being as suggested by Schalock and Verdugo (2002), despite their wide recognition in the field of psychology and mental health. Generally, these measures have been incorporated into welfare economic models to evaluate the cost-effectiveness of psychosocial interventions, trying to maximize their usefulness for as many people as possible, without being the best metrics to represent non-health benefits (Jones et al., 2020). Nevertheless, according to Owen et al. (2015) open dialogue with stakeholders shows their experiences and the changes in their lives better than the use of standardized questionnaires, which would explain its little uptake by organizations.

Regarding the use of proxies that allow the monetization of the quantified value, according to Akingbola et al. (2015) social organizations often follow a commercial logic rather than a non-profit logic and value the benefits using revealed preference methods based in market prices. In some cases, these types of proxies have been used in the reviewed studies (e.g., cost of renting a one bedroom flat or cost of a life insurance payout). However, the proxies that are more commonly applied are in line with Fujiwara (2014) proposal and generally refer to the amount of money that would produce an equivalent effect through psychological care or therapy services, for example.

Our results corroborate the use of SROI as the main methodology in the field of social accounting. Corvo et al. (2021) conclude that of the 98 impact assessment models identified in the literature, SROI is the most widely used compared to other models coming from performance and management systems. Furthermore, the SROI methodology has emerged as a preferred technique for measuring social value and impact in the field of health and care services (Krlev et al., 2013), since some organizations such as the UK Department of Health have been promoting its application in social accounting for more than a decade (Millar and Hall, 2013; Banke-Thomas et al., 2015; Ashton et al., 2020; Corvo et al., 2021). However, this is not surprising since we collected the analyzed reports from the Social Value UK database, which could lead to a biased result.

The conducted analysis has revealed the difficulty in measuring the emotional value of disability organizations, given its subjective, intangible and multidisciplinary nature. However, there is considerable consensus regarding the need to recognize emotional well-being as part of social accounting, moving from a financial accounting system focused on inputs and tangible benefits to a social accounting model aligned with the components of the outcome (Van Loon et al., 2013).

The dispersion of instruments and methodologies suggest the need to harmonize the measurements to improve the comparability and credibility of the analysis. This study contributes to identifying some of the most common practices among disability organizations that could be the basis of a framework for the development of a common methodology applicable to the disability sector. The relevance of the use of social accounting methodologies for disability organizations is clear from the fact that studies affirm their importance not only in setting strategies and selecting alternatives, but also in improving effectiveness and efficiency and in the internal and external communication, coinciding with conclusions reached by Schalock et al. (2008).

Our study has implications for the management of disability organizations that can use social accounting to evaluate their performance and improve their effectiveness and efficiency, showing a more complete picture of the social value generated. Likewise, it can be an instrument to make the contribution and social benefits of these organizations visible in all their breadth, improving transparency and legitimacy.

Since we have confirmed the difficulty in standardizing emotional value and the enormous dispersion of instruments for its assessment, future lines of research could propose a homogeneous social accounting model based on an empirical study of the emotional value drivers in disability organizations. The measurement framework should be in line with the existing psychological scales on quality of life and emotional well-being that are actually used by disability organizations in their interventions, adding the emotional value assessment into their social accounting. This framework would facilitate the standardization and comparability of the performance results of disability organizations. Likewise, future research could investigate the usefulness of this social accounting model in management. Just as previous studies have proven the relevance of generic social accounting in multiple organizational functions such as strategic planning or marketing, there is still a need to discover the contribution that the measurement of emotional value in those areas.

## Data Availability Statement

The original contributions presented in the study are included in the article, further inquiries can be directed to the corresponding author/s.

## Author Contributions

PT-V, SA, and VF-R contributed to the design of the research, to the analysis of the results, and to the writing of the manuscript. The authors had an equal contribution to this research. All authors contributed to manuscript revision. All authors read and approved the final manuscript.

## Author Disclaimer

All views expressed in the paper, and any omissions or errors therein, are those of the author.

## Conflict of Interest

The authors declare that the research was conducted in the absence of any commercial or financial relationships that could be construed as a potential conflict of interest.

## Publisher's Note

All claims expressed in this article are solely those of the authors and do not necessarily represent those of their affiliated organizations, or those of the publisher, the editors and the reviewers. Any product that may be evaluated in this article, or claim that may be made by its manufacturer, is not guaranteed or endorsed by the publisher.

## Appendix: Analyzed studies

**Table:**

1	Akingbola, K., Phaetthayanan, S., & Brown, J. (2015). A-Way Express Courier: Social Enterprise and Positive Psychology. Nonprofit Management and Leadership, 26(2), 173-188.
2	Care Network (2012) Valuing Care Network: A SROI Framework and Forecast Analysis for Care Network (Blackburn with Darwen) Ltd. Care Network. https://socialvalueuk.org/wp-content/uploads/2016/03/Care%20Network%20SROI%20Report%20accredited%20final%20version.pdf [Accessed April 7, 2021].
3	Chen, C. (2018). SROI Report of the “Job Design Support by Collaborators for Disabled People in Open Job Market” project. Taiwan NPO Self-Regulation Alliance. https://socialvalueuk.org/wp-content/uploads/2019/02/The-SROI-Report-of-JSCDO-.pdf [Accessed April 7, 2021].
4	Craemer, R. (2011). A social cost-benefit analysis: Early intervention programs to assist children with hearing loss develop spoken language. First Voice. https://socialvalueuk.org/wp-content/uploads/2016/03/First%20Voice%20CBA_Final%20Report%20August%202011.pdf [Accessed April 7, 2021].
5	Deloitte (2013). Economic impact of social care services: Assessment of the outcomes for disabled adults with moderate care needs, Final Report. Deloitte. https://socialvalueuk.org/wp-content/uploads/2016/03/Deloitte-report.pdf [Accessed April 7, 2021].
6	Durie, S. (2009). Community First (Moray) Social Return on Investment (SROI) Analysis. An evaluation of The Handyperson Service (Moray) social value creation. Community First (Moray). https://socialvalueuk.org/wp-content/uploads/2016/05/community-first-moray-sroi.pdf [Accessed April 7, 2021].
7	Farr, M., & Cressey, P. (2019). The social impact of advice during disability welfare reform: from social return on investment to evidencing public value through realism and complexity. Public Management Review, 21(2), 238-263.
8	Forth Sector Development and PricewaterhouseCoopers (2011). Social Return on Investment (SROI) Analysis: An evaluation of social added value of the employability pilot. Registers of Scotland and Haven Products. https://socialvalueuk.org/wp-content/uploads/2016/09/Public-Social-Partnerships-Employability-Pilot-SROI.pdf [Accessed April 7, 2021].
9	Gardner C., and Isard, P. (2014). Ability not disability: Arts, bravery and changing views in Ireland. Blue Teapot. https://socialvalueuk.org/wp-content/uploads/2017/02/Blue-Teapot-SROI-FINAL.pdf [Accessed April 7, 2021].
10	Gauge NI (2012) The Cedar Foundation Community Inclusion Programmes SROI Study April 2007-April 2012. The Cedar Foundation. https://socialvalueuk.org/wp-content/uploads/2016/03/The%20Cedar%20Foundation%20Community%20Inclusion%20Programmes%20SROI.pdf [Accessed April 7, 2021].
11	Golden Lane Housing (2014). Social Impact Report 2014. Golden Lane Housing. https://socialvalueuk.org/wp-content/uploads/2016/03/GLH_SocialImpactReport_Oct14_21.pdf [Accessed April 7, 2021].
12	Golics, C. J., Basra, M. K. A., Finlay, A. Y., & Salek, S. (2013). The impact of disease on family members: a critical aspect of medical care. Journal of the Royal Society of Medicine, 106(10), 399-407.
13	Hamelin Trust (2011). The Value of Hamelin Trust's Roots and Shoots: An SROI Analysis. Hamelin Trust. https://socialvalueuk.org/wp-content/uploads/2016/03/Roots%20and%20Shoots%20FINAL.pdf [Accessed April 7, 2021].
14	Hoeber, A. (2011) SROI Evaluative Analysis of Introduction to Supported Employment Program. Saskatchewan Abilities Council. https://socialvalueuk.org/wp-content/uploads/2016/03/SOCIAL%20RETURN%20ON%20INVESTEMENT%20REPORT%20final%2007%2030%20%202012%20TVB.pdf [Accessed April 7, 2021].
15	Hutchinson, C., Berndt, A., Cleland, J., Gilbert-Hunt, S., George, S., & Ratcliffe, J. (2020). Using social return on investment analysis to calculate the social impact of modified vehicles for people with disability. Australian Occupational Therapy Journal, 67(3), 250-259.
16	Jones, C., Windle, G., & Edwards, R. T. (2020). Dementia and imagination: A social return on investment analysis framework for art activities for people living with dementia. The Gerontologist, 60(1), 112-123.
17	Leck, C. (2012). Social Return on Investment (SROI) Evaluation Report of The Houghton Project. Care Farming West Midlands. https://socialvalueuk.org/wp-content/uploads/2016/03/Houghton%20Project%20SROI%20assured.pdf [Accessed April 7, 2021].
18	MARCAL Consulting (2014). Birmingham Disability Resource Centre: A Social Return on Investment Analysis. Disability Resource Centre (DRC). https://socialvalueuk.org/wp-content/uploads/2016/07/DRC-SROI-report.pdf [Accessed April 7, 2021].
19	McCorriston, E. (2015). SROI Evaluative Analysis: Realise Futures Social Businesses .Realise Futures. https://socialvalueuk.org/wp-content/uploads/2016/03/Realise%20Futures%20SROI%20Analysis%20Final%20June%202015%20Social%20Value%20UK.pdf [Accessed April 7, 2021].
20	Minney, H. (2011). Social Return on Investment (SROI) on Quality Checkers (Skills for People). Minney.org Ltd. https://socialvalueuk.org/wp-content/uploads/2016/03/SROI_QC_Report_Assured.pdf [Accessed April 7, 2021].
21	Nicol, J. (2011). The Breadmaker Social Return on Investment Evaluation Report. Aberdeen Day Project Ltd. https://socialvalueuk.org/wp-content/uploads/2016/03/The%20Breadmaker%20SROI%20April%202011.pdf [Accessed April 7, 2021].
22	Novikova, I. (2016). Evaluating the impact of the “Status: Online” programme in Russia, “CAF” Foundation for Philanthropy Support and Development. https://socialvalueuk.org/wp-content/uploads/2017/02/SROI-Report-SOP-certified-4-January.pdf [Accessed April 7, 2021].
23	Onyx, J., Darcy, S., Grabowski, S., Green, J., & Maxwell, H. (2018). Researching the social impact of arts and disability: Applying a new empirical tool and method. VOLUNTAS: International Journal of Voluntary and Nonprofit Organizations, 29(3), 574-589.
24	Owen, F., Li, J., Whittingham, L., Hope, J., Bishop, C., Readhead, A., & Mook, L. (2015). Social return on investment of an innovative employment option for persons with developmental disabilities: Common ground co-operative. Nonprofit Management and Leadership, 26(2), 209-228.
25	Ruiz-Lozano, M., Tirado-Valencia, P., Sianes, A., Ariza-Montes, A., Fernández-Rodríguez, V., & López-Martín, M. C. (2020). SROI methodology for public administration decisions about financing with social criteria. A case study. Sustainability, 12(3), 1070.
26	Shaw, A. (2018). Using the Social Return on Investment Framework to Evaluate Behavior Changes of Individuals Living With Learning Difficulties. Social Marketing Quarterly, 24(4), 281-298.
27	Sital-Singh, P. (2011) RNIB and Action for Blind People Social Firms: A Social Impact Measurement. RNIB and London South Bank University. https://socialvalueuk.org/wp-content/uploads/2016/03/RNIB_and_Action_Social_Firms_Impact_Measurement_Final_Report_v1.0_External_20.9.2011_merged[1].pdf [Accessed April 7, 2021].
28	The Action Group (2011) Social Return on Investment (SROI) Analysis: An evaluation of social added value for Real Jobs. The Action Group, Edinburgh. https://socialvalueuk.org/wp-content/uploads/2016/03/SROI%20Real%20Jobs%20Evaluation_.pdf [Accessed April 7, 2021].
29	Willis, E., Semple, A. C., & de Waal, H. (2018). Quantifying the benefits of peer support for people with dementia: A Social Return on Investment (SROI) study. Dementia, 17(3), 266-278.
